# Liver dysfunction is associated with poor prognosis in patients after immune checkpoint inhibitor therapy

**DOI:** 10.1038/s41598-020-71561-2

**Published:** 2020-09-02

**Authors:** Keisuke Yokohama, Akira Asai, Masahiro Matsui, Norio Okamoto, Hidetaka Yasuoka, Tomohiro Nishikawa, Hideko Ohama, Yusuke Tsuchimoto, Yoshihiro Inoue, Shinya Fukunishi, Kazuhisa Uchiyama, Kazuhide Higuchi

**Affiliations:** 1 Second Department of Internal Medicine, Osaka Medical College, 2-7, Daigakumachi, Takatsuki, Osaka 569-8686 Japan; 2 Internal Medicine, Hokusetsu General Hospital, Takatsuki, Japan; 3 Department of General and Gastroenterological Surgery, Osaka Medical College, Takatsuki, Japan

**Keywords:** Cancer, Cancer immunotherapy

## Abstract

Immune-related adverse events (irAEs) are induced by immune checkpoint inhibitors (ICIs). Liver is one of the main target organs which irAEs occur and we investigated the influence of liver dysfunction on prognosis of patients after ICIs. From July 2014 to December 2018, 188 patients with diverse cancers who received ICIs (nivolumab or pembrolizumab) were enrolled. Twenty-nine patients experienced liver dysfunction of any grades after ICIs. Progression-free survival (PFS) was significantly shorter in the liver dysfunction-positive group than in the liver dysfunction-negative group, and a similar result was obtained for Overall survival (OS). Multiple logistic regression analysis revealed liver metastasis and alanine aminotransferase before ICIs were associated with a higher incidence of liver dysfunction after ICIs. Regardless of liver metastasis, PFS and OS were significantly shorter in the liver dysfunction-positive group. In conclusion, this study suggests liver dysfunction is associated with poor prognosis in patients after ICIs with diverse cancers.

## Introduction

Cancer was the second most common cause of death in the last decade, and was responsible for an estimated 9.6 million deaths worldwide in 2018^1^. Cancer is treated by surgical resection, radiation and chemotherapy, but the mortality rate in cancer patients is still high. Therefore, new strategies to treat cancer are needed.

Recently, immunotherapy has become a mainstay of treatment of cancer. Antibodies against cytotoxic T-lymphocyte antigen 4 (CTLA-4) and programmed cell death protein 1 (PD-1) and its ligands (PD-L1/PD-L2) can modulate the immune response to cancer clearance in a various human malignancies. The PD-1 pathway operates in the tumor microenvironment, unlike CTLA-4, which mainly works in the lymph nodes^2^. PD-1 interacts with its ligands on the surface of tumor cells and tumor-associated macrophages, dendritic cells, fibroblasts, and activated T cells in the immune milieu^3–6^ . The binding of PD-1 with its ligands block antitumor activity of T cell^7–9^. Nivolumab and pembrolizumab are fully humanized immunoglobulin G4 PD-1 immune checkpoint inhibitor (ICI) antibodies that selectively block the interaction of the PD-1 receptor and its ligands^10,11^. These inhibitors have significant clinical activity and have improved prognosis in multiple cancer types, including non-small-cell lung cancer, renal cell carcinoma, urothelial carcinoma, gastric cancer, head and neck squamous cell carcinoma, malignant melanoma, and hepatocellular carcinoma^11–21^.

Blocking immunosuppressive ligand receptor interactions enhances the anticancer effects of lymphocytes. However, these molecules are also involved in healthy immune tolerance, and therefore adverse reactions to self-antigens can occur. The adverse events caused by autoimmune reactions are currently denoted as immune-related adverse events (irAEs) to differentiate them from idiosyncratic drug-induced organ damage ^22^.

Most patients with irAEs can be managed, but some patients treated with ICIs have died because of irAEs^23^. Conversely, some patients treated with ICIs who develop irAEs have higher autoimmune responses and the drugs have higher efficacy^24–26^. Therefore, irAEs are a major problem with ICI therapies, and the influence of irAEs in patients receiving ICI therapies is unclear.

The liver is one of the organs that potentially manifests irAEs, and it was reported that immune-mediated liver dysfunction of any grade occurs in 1–8% of patients with PD-1 inhibitors^27–29^. There is no report on association between liver dysfunction due to irAE and prognosis in patients treated with ICIs.

In this multicenter retrospective cohort study of patients treated with ICI monotherapy for diverse cancers, we investigated the influence of liver dysfunction on prognosis in patients after ICI treatment.

## Results

### Patient characteristics

**Figure 1:**
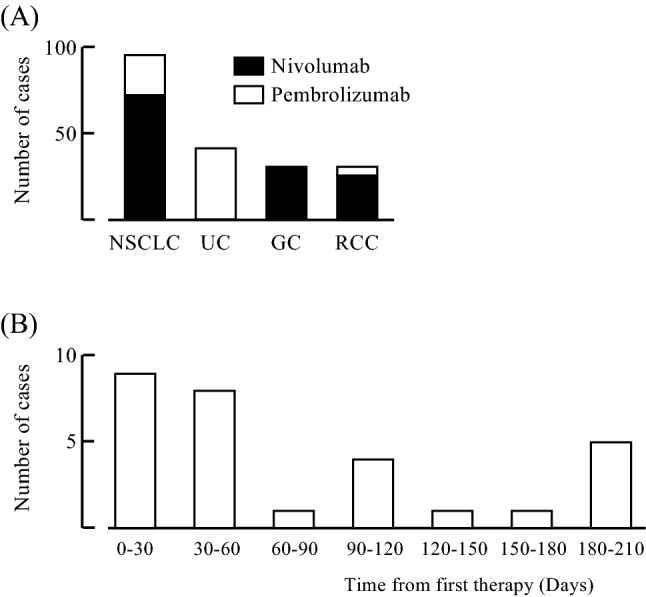
**(A)** Number of patients with different cancer types. *NSCLC* non-small cell lung cancer; *UC* urothelial cancer, *GC* gastric cancer; *RCC* renal cell carcinoma). **(B)** Time from first therapy to onset of liver dysfunction.

We included 188 patients with advanced cancer treated with ICIs monotherapy (nivolumab or pembrolizumab) (Fig. 1A). There were no patients treated with ICI combination therapy. The baseline clinical characteristics for the study cohort at initiation of ICIs are summarized in Table 1. The majority of patients (72.9%) were over 65 years old. Fifty-five patients (29.3%) had liver diseases; most were in the remission phase of HBV infection, and their liver functions before ICIs were normal. Thirty patients (16.0%) had liver metastases before the initiation of ICIs and their liver functions were also normal before initiation of ICIs. ICIs were selected as second-line or later-line treatments in the majority of patients. However, 13 patients (6.4%; 12 non-small cell lung cancer patients and 1 urothelial carcinoma patient) received ICIs as first-line treatment. Only 4 patients (2.1%) had been previously treated with ICIs.

**Table 1:** Baseline characteristics of patients receiving ICIs (n = 188).

Male/female	140/48
Age (years), median (range)	71.5 (36–86)
Patients over 65 years old	137 (72.9%)
Body weight (kg)	56.3 ± 11.7
Patients with liver disease	55 (29.3%)
Patients with liver metastasis	30 (16.0%)
**Number of previous lines of chemotherapy**	
0 (first-line)	12 (6.4%)
1 (second-line)	79 (42.0%)
≥ 2 (third- or later line)	97 (51.6%)
Previously received ICIs	4 (2.1%)
White blood cell count (/μL)	6,813 ± 3,583
Hemoglobin (g/dL)	11.1 ± 1.8
Platelet count (× 10^4^/μL)	27.0 ± 11.5
Lymphocyte count (/μL)	1,253 ± 655
Blood urea nitrogen (mg/dL)	18.1 ± 7.5
Blood creatinine (mg/dL)	1.06 ± 0.76
eGFR (mL/min/1.73m^2^)	61.4 ± 23.2
Total bilirubin (mg/dL)	0.49 ± 0.30
AST (IU/L)	23.4 ± 12.9
ALT (IU/L)	16.7 ± 13.4
PT (%)	93.5 ± 15.0
Patients with anti-nuclear antibodies	40 (37.7%)
Rheumatoid factor (IU/mL)	10.9 ± 17.2

*AST* aspartate aminotransferase; *ALT* alanine aminotransferase; *eGFR* estimate glomerular filtration rate; *ICI* immune checkpoint inhibitor; *PT* prothrombin time.

### Frequency and severity of liver dysfunction

Twenty-nine of 188 (15.4%) patients developed liver dysfunction of any grade after ICIs (Table 2). Seventeen percent of patients treated with nivolumab developed liver dysfunction and 13% of patients treated with pembrolizumab developed liver dysfunction. Ten patients (5.3%) required the interruption of ICIs (dose delay, cessation, or therapeutic intervention for immunosuppressive therapy) due to grade 2 or more of liver dysfunction after ICIs. The frequency of interruption due to severe liver dysfunction in patients treated with pembrolizumab (9.8%) was higher than that in patients treated with nivolumab (3.1%). The median time to onset of liver dysfunction after ICIs was 43 days (range 7–210 days) (Fig. 1B). Most cases of liver dysfunction occurred within 3 months of the initiation of the ICI therapy, although five cases occurred more than half a year after initiation.

**Table 2:** Frequency and severity of liver dysfunction after ICI monotherapy.

	All Grade	Grade 1	Grade 2	Grade 3	Grade 4
All cases (n = 188)	15.4% (n = 29)	10.1% (n = 19)	1.6% (n = 3)	3.2% (n = 6)	0.5% (n = 1)
Nivolumab (n = 127)	17.1% (n = 21)	13.4% (n = 17)	0.8% (n = 1)	1.6% (n = 2)	0.8% (n = 1)
Pembrolizumab (n = 61)	13.1% (n = 8)	3.3% (n = 2)	3.3% (n = 2)	6.6% (n = 4)	0% (n = 0)

*ICI* immune checkpoint inhibitor.

### Prognosis of patients treated with ICIs

**Figure 2:**
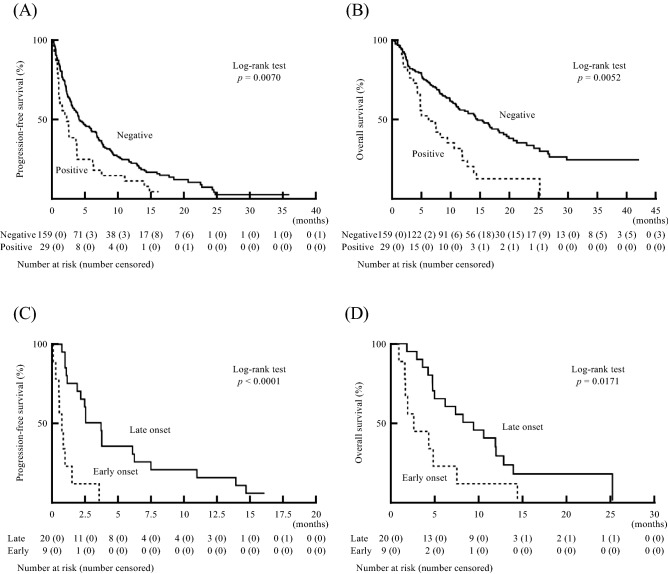
**(A, B)** The influence of liver dysfunction on PFS **(A)** and OS **(B)** in patients with diverse cancer types after ICI treatment (unadjusted data). **(C, D)** Comparison of PFS **(C)** and OS **(D)** based on early onset and late onset of liver dysfunction after ICI treatment (unadjusted data). *PFS* progression-free survival; *OS* overall survival; *ICI* immune checkpoint inhibitor).

We compared the prognosis of patients with liver dysfunction (positive group) and without liver dysfunction (negative group). The Progression-free survival (PFS) in the positive group (median 64 days, 95% CI 28–110 days) was significantly shorter than that in the negative group (median: 121 days, 95% CI 89–178 days) (Fig. 2A). Additionally, the Overall survival (OS) in the positive group (median 184 days, 95% CI 126–316 days) was significantly shorter than that in the negative group (median: 427 days, 95% CI 328–548 days) (Fig. 2B). We further subdivided patients in the positive group based on time to liver dysfunction: patients who developed liver dysfunction within 30 days after ICI therapy were defined as the early onset group, and patients who developed liver dysfunction more than 30 days after ICI therapy were defined as the late onset group. The PFS in the early onset group (median 21 days, 95% CI 1–44 days) was significantly shorter than that in the late onset group (median: 93 days, 95% CI 33–186 days) (Fig. 2C). The OS in the early onset group (median 76 days, 95% CI 25–223 days) was also significantly shorter than that in the late onset group (median: 263 days, 95% CI 141–358 days) (Fig. 2D). In conclusion, the PFS and OS of the positive group were significantly shorter than those of the negative group, and among patient with liver dysfunction, those with early onset had a worse prognosis than those with late onset.

### Predictive factors of liver dysfunction after ICIs

We investigated which factors were associated with liver dysfunction after ICI. For univariate screening, univariate analyses were performed and then those risk factors deemed to have a statistically significant association with the outcome in the univariate analyses were then included in the multiple logistic regression model. Baseline clinical characteristics between the positive group and the negative group were compared. In univariate analysis, there were significant differences in liver metastasis (*p* = 0.0014), hemoglobin (*p* = 0.0439), and alanine aminotransferase (ALT) (*p* = 0.0085) (Table 3A). Multiple logistic regression analysis revealed that liver metastasis (OR 3.24, 95% CI 1.27–8.30,* p* = 0.0161) and ALT (> 13 IU/L vs ≤ 13 IU/L; OR 2.54, 95% CI 1.08–5.96,* p* = 0.0294) before ICIs were significantly associated with a higher incidence of liver dysfunction after ICIs (Table 3B).

**Table 3:** Characteristics of patients with and without liver dysfunction after ICIs.

(A) Univariate analysis	Positive group (n = 29)	Negative group (n = 159)	*p* value
Male/female	23/6	117/42	0.5076
Age (years), median (range)	69 (43–86)	72 (36–85)	0.2359
Patients over 65 years old	19 (58.6%)	120 (75.5%)	0.0697
Body weight (kg)	56.8 ± 10.4	56.2 ± 12.0	0.7874
Patients with liver disease	9 (31.0%)	46 (28.9%)	0.8197
Patients with liver metastasis	11 (37.9%)	19 (11.9%)	0.0014
**Number of previous lines of chemotherapy**			0.4092
2 > (First- or second-line)	12 (41.4%)	79 (49.7%)	
≥ 2 (third- or later line)	17 (58.6%)	80 (50.3%)	
Previously received ICIs	0 (0%)	4 (2.5%)	0.2442
White blood cell count (/μL)	6,776 ± 3,078	6,819 ± 3,676	0.9513
Hemoglobin (g/dL)	10.5 ± 1.7	11.2 ± 1.9	0.0485
Platelet count (× 10^4^/μL)	25.7 ± 10.7	27.2 ± 11.7	0.4970
Lymphocyte cell (/μL)	1,201 ± 458	1,262 ± 686	0.6373
Blood urea nitrogen (mg/dL)	18.6 ± 5.5	18.0 ± 7.9	0.6784
Blood creatinine (mg/dL)	1.00 ± 0.28	1.07 ± 0.81	0.6029
eGFR (mL/min/1.73m^2^)	60.0 ± 19.3	61.6 ± 23.9	0.7238
Total bilirubin (mg/dL)	0.55 ± 0.27	0.48 ± 0.31	0.2782
AST(IU/L)	27.5 ± 16.3	22.6 ± 12.2	0.0850
ALT(IU/L)	23.3 ± 19.9	15.5 ± 11.5	0.0105
PT (%)	90.0 ± 13.0	94.2 ± 15.3	0.1998
Patients with anti-nuclear antibodies	7 (36.8%)	7 (37.9%)	0.8405
Rheumatoid factor (IU/mL)	13.5 ± 20.9	10.4 ± 16.3	0.5198

(B) Multivariate analysis	OR	95% CI	*p* value
Liver metastasis	3.54	1.35–9.23	0.0099
Hemoglobin (≤ 11.6 g/dL)	1.98	0.80–4.88	0.1376
ALT (> 13 IU/L)	2.54	1.07–6.05	0.0345

(A) Univariate analysis: *AST* aspartate aminotransferase; *ALT* alanine aminotransferase; *eGFR* estimate glomerular filtration rate; *ICI* immune checkpoint inhibitor; *PT* prothrombin time.

(B) Multivariate analysis: *OR* odds ratio; *CI* confidence interval; *ALT* alanine aminotransferase; *N.R.* not reported.

### Influence of liver dysfunction after ICIs on prognosis based on liver metastasis

**Figure 3:**
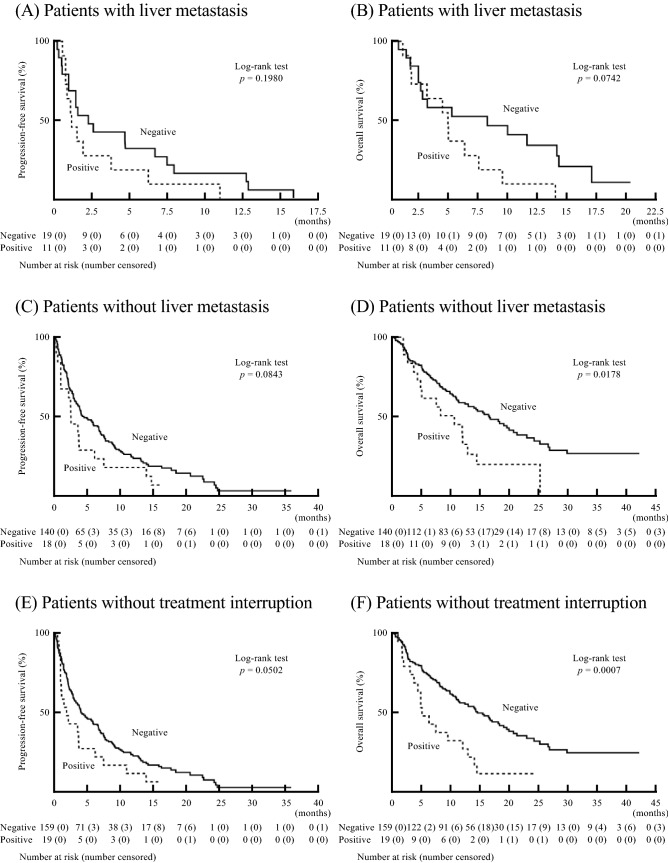
**(A, B)** The influence of liver dysfunction on PFS **(A)** and OS **(B)** in patients with diverse cancer types with liver metastasis after ICI treatment (unadjusted data). **(C, D)** The influence of liver dysfunction on PFS **(C)** and OS **(D)** in patients with diverse cancer types without liver metastasis after ICI treatment (unadjusted data). **(E, F)** The influence of liver dysfunction on PFS **(E)** and OS **(F)** in patients with diverse cancer types who did not experience interruption of ICI treatment (unadjusted data). *PFS* progression-free survival; *OS* overall survival; *ICI* immune checkpoint inhibitor.

Next, the effect of liver dysfunction on the prognosis of patients with liver metastasis was investigated. Among patients with liver metastasis, the PFS of patients with liver dysfunction (median 33 days, 95% CI 21–112 days) was shorter than that of patients without liver dysfunction (median: 67 days, 95% CI 28–200 days) (Fig. 3A). The OS of patients with liver dysfunction (median 141 days, 95% CI 45–220 days) was also shorter than that of patients without liver dysfunction (median: 242 days, 95% CI 65–421 days) (Fig. 3B). Similar results were observed among patients without liver metastasis. Among patients without liver metastasis, the PFS of patients with liver dysfunction (median: 75 days, 95% CI 28–112 days) was shorter than that of patients without liver dysfunction (median: 122 days, 95% CI 98–201 days) (Fig. 3C). The OS of patients with liver dysfunction was also significantly shorter (median 281 days, 95% CI 126–384 days) than that of patients without liver dysfunction (median: 492 days, 95% CI 339–620 days) (Fig. 3D). These results indicated that the prognosis of patients with liver dysfunction was poor regardless of liver metastasis.

### Influence of liver dysfunction after ICIs on prognosis of patients without treatment interruption

There were 10 patients who required interruption of ICI treatment (dose delay, cessation, or therapeutic intervention for immunosuppressive therapy) due to grade 2 or more liver dysfunction. No patient died of liver failure. Based on the guidelines for liver dysfunction in the CTCAE 4.0, when patients had grade 1 liver dysfunction after ICIs, treatment could be continued with close monitoring. Therefore, the prognoses of patients with liver dysfunction who continued ICI treatment were studied. Among patients who did not experienced interruption of ICIs, the PFS of patients with liver dysfunction (median 56 days, 95% CI 28–112 days) was shorter than that of patients without liver dysfunction (median: 121 days, 95% CI 89–178 days) (Fig. 3E). Similarly, the OS of patients with liver dysfunction was significantly shorter (median 148 days, 95% CI 87–358 days) than that of patients without liver dysfunction (median: 427 days, 95% CI 328–548 days) (Fig. 3F). Therefore, the prognosis of patients who experienced liver dysfunction after ICIs was poor regardless of whether they had to discontinue ICIs.

## Discussion

Although, there are several reports on predictors of irAEs after ICI therapy^30,31^, no report has focused on liver dysfunction. This study is the first report to reveal that liver metastasis before ICI therapy is a predictive factor for liver dysfunction after ICI therapy. The frequency of liver dysfunction after ICI therapy was higher in patients with liver metastases than in patients without liver metastases, and the odds ratio was 3.24 (95% CI 1.27–8.30). We also found that, among patients without liver metastases, the prognosis of patients with liver dysfunction after ICI therapy was clearly worse than that of patients without liver dysfunction. Therefore, patients with liver dysfunction after ICI therapy have poorer prognosis than patients without liver dysfunction.

The precise pathophysiology underlying immune-related adverse events is unknown but is believed to be related to the role that immune checkpoints play in maintaining immunologic homeostasis. As liver dysfunction after ICI therapy cannot be explained by any of the four mechanisms of irAE development, we have identified four potential mechanisms for liver dysfunction after ICI therapy. The first is increased T cell activity against antigens that are present in tumors and healthy tissue. This type of liver dysfunction is thought to be the most common type of irAE. The second is increased levels of preexisting autoantibodies^32^. This type of liver dysfunction is caused by autoimmune toxicities and includes neuromuscular dysfunction, Guillain–Barre syndrome, autoimmune thyroiditis, and acute presentation of AIH. The third is drug–induced liver injury. This type is due to immune-mediated or hypersensitive drug reaction or exposure to toxic doses of ICIs. The fourth potential mechanism is liver metastasis. This type sometimes occurs due to tumor progression even if the patient receives ICIs.

Previous studies have reported frequencies of all-grade and grade 3–4 liver dysfunction of 1.0–7.6% and 0.5–2.3%, respectively, in patients treated with nivolumab for malignant melanoma^27,28^. In a phase II/III trial of patients treated with pembrolizumab for non-small cell lung cancer, the frequency of all-grade liver dysfunction was 2.0–4.7%, and that of grade 3 or 4 liver dysfunction was 0.3–0.6%^29^. In this study, liver dysfunction of any grade after ICI monotherapy occurred in more than 15% of patients. Furthermore, 5.3% of patients required interruption of ICI monotherapy due to grade 3 or 4 severe liver dysfunction. We believe that the high frequency of liver dysfunction after ICI therapy in this study was caused by including not only irAE but also other types such as drug–induced liver injury. Liver biopsy will be required to determine the type of liver dysfunction in patients treated with ICIs.

The PFS and OS in patients who developed early-onset liver dysfunction were significantly shorter than those with late-onset liver dysfunction in our study. There are many reports about the relationship between the onset time of the irAE and prognosis^33–35^. Cortellini et al. reported that the early onset of liver dysfunction might be considered a treatment-related effect of cytokine release syndrome or hypersensitivity to the drug^34^. Another report suggests that the unidentified immune activity in the tumor may enhanced the effect of nivolumab in the early phase, which results in T cell recognition and activity against antigens in healthy tissues provide improving treatment with ICIs and important clues on the mechanism of PD-1-mediated toxicity and antitumor efficacy^35^. Further research is required to elucidate the mechanisms driving these associations.

This study suggests that liver dysfunction is associated with poor prognoses of patients receiving ICI therapy against multiple cancer types. There might be a selection bias in patient selection and collecting the patient information, because this study was retrospective with a small number of non-randomized, medical record-based cases. Therefore, in order to further clarify the influence of liver dysfunction after ICI therapy on the prognosis of patients with ICI therapy, it is necessary to continue to accumulate more cases and investigate these questions prospectively.

## Methods

### Accordance and guideline

All procedures performed in this study were in accordance with the ethical standards of the institution and ethical guideline for medical and human subject in Japan and with the 1964 Helsinki declaration and its later amendments.

### Study design and participants

One hundred and eighty-eight patients with advanced-stage cancer (95 non-small cell lung cancer patients, 38 urothelial carcinoma patients, 28 gastric cancer patients and 27 renal cell carcinoma patients) treated with ICIs at two study centers (Osaka Medical College Hospital and Hokusetsu General Hospital) from July 2014 to December 2018 were enrolled in this study. All patients were treated with nivolumab or pembrolizumab monotherapy. We retrospectively collected the following patient data from medical records: age, sex, weight, stage of cancer, the number of previous chemotherapy lines, and laboratory data before the initiation of ICI therapy. ICIs induce various adverse events, which are graded according to the Common Terminology Criteria for Adverse Events, version 4.0 (CTCAE 4.0), a tool commonly used for the evaluation of adverse events of chemotherapy. We treated adverse events according to the clinical guidelines of the American Society of Clinical Oncology^36^. We used these data to investigate the influence of liver dysfunction on prognosis in these patients after ICI therapy.

### Statistical analysis

PFS was calculated as the time from the initiation of ICI therapy until tumor progression as determined by the treating physician, death from any cause, or last follow-up, whichever occurred first. OS was calculated from the time of the initiation of ICI therapy until death from any cause or last follow-up. We used the Kaplan–Meier method and log-rank test to compare the prognosis (PFS and OS) of patients with liver dysfunction and without liver dysfunction^37^. Figures 2 and 3 used the unadjusted data. And we performed the matching to adjust for the potential confounders using propensity score (Supplemental Fig. 1). Matching was performed with the use of a 1:1 matching protocol without replacement, with a caliper width equal to 0.05 of the standard deviation of the logit of the propensity score.

The clinical laboratory values were not normally distributed; therefore, the Mann–Whitney U test was used to analyze continuous scales. The Fisher’s exact test was used to analyze the nominal scales. For univariate screening, univariate analyses were performed. Then those risk factors deemed to have a statistically significant association with the outcome in the univariate analyses were then included in the multiple logistic regression model. We also analyzed using the forward–backward stepwise method, and the same risk factors were extracted. All recorded *p* values were two-sided, and differences with *p* < 0.05 were considered significant. All analyses were performed using JMP software, version 13 (SAS Institute Inc., Cary, NC, USA)^38^.

### Ethics statement

The study was approved by the Institutional Review Board of the Osaka Medical College (IRB approval number: 2126). Informed consent was obtained in the form of opt-out and patients who rejected were excluded. 

## Supplementary information

Supplementary information
