# Supplementary material for: Liver dysfunction is associated with poor prognosis in patients after immune checkpoint inhibitor therapy
Source: Sci Rep. 2020 Sep 2;10:14470. doi: 10.1038/s41598-020-71561-2 (PMC7468148; doi:10.1038/s41598-020-71561-2)
Supplement: Supplementary file 1 — Supplementary information [file 41598_2020_71561_MOESM1_ESM.pptx]

## Slide 1
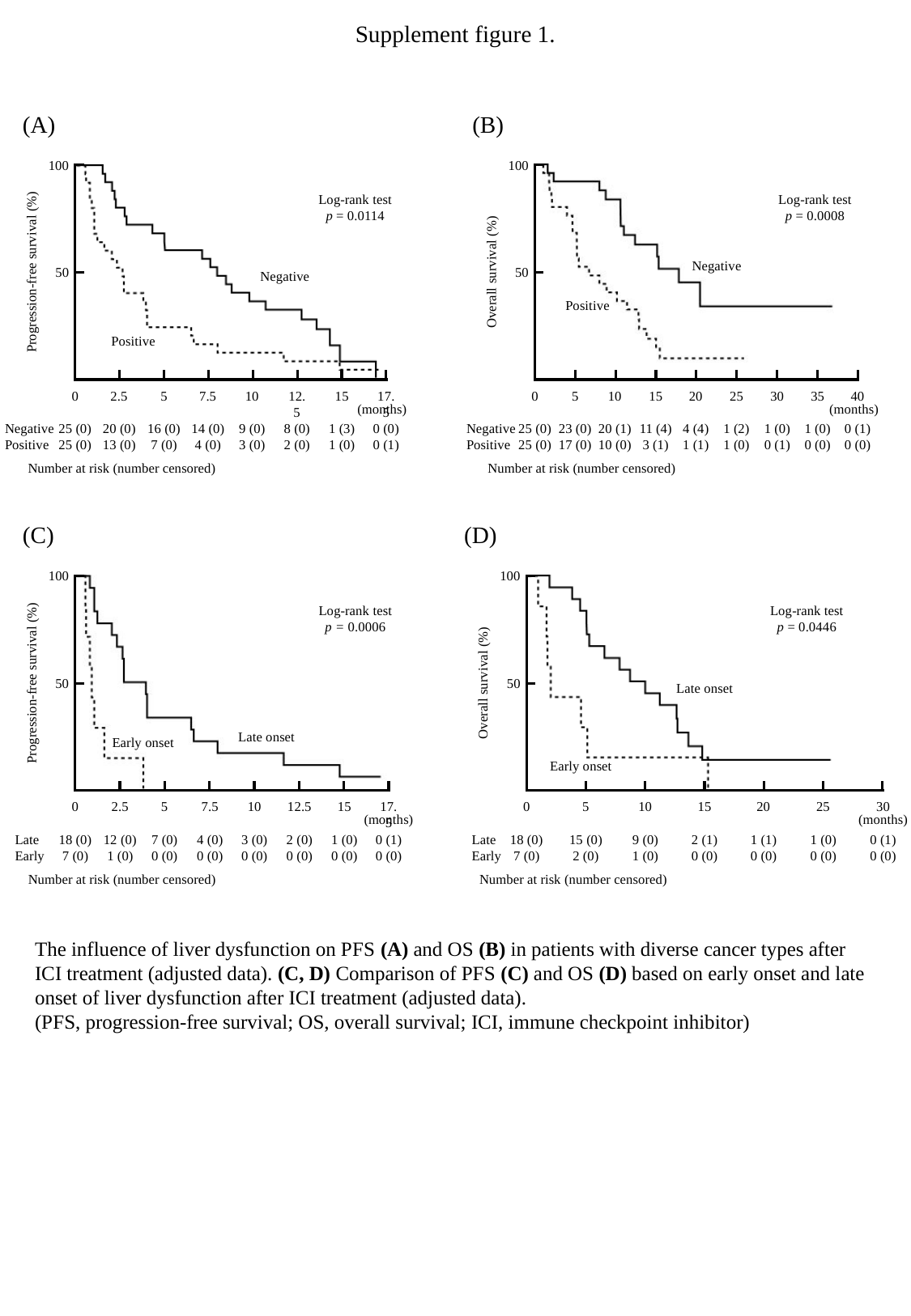

Supplement figure 1.
(A)
(B)
100
100
Log-rank test
p = 0.0114
Log-rank test
p = 0.0008
Negative
Progression-free survival (%)
Overall survival (%)
50
50
Negative
Positive
Positive
0
2.5
5
7.5
10
12.5
15
17.5
0
5
10
15
20
25
30
35
40
(months)
(months)
Negative
Positive
25 (0)
25 (0)
20 (0)
13 (0)
16 (0)
7 (0)
14 (0)
4 (0)
9 (0)
3 (0)
8 (0)
2 (0)
1 (3)
1 (0)
0 (0)
0 (1)
Negative
Positive
25 (0)
25 (0)
23 (0)
17 (0)
20 (1)
10 (0)
11 (4)
3 (1)
4 (4)
1 (1)
1 (2)
1 (0)
1 (0)
0 (1)
1 (0)
0 (0)
0 (1)
0 (0)
Number at risk (number censored)
Number at risk (number censored)
(C)
(D)
100
100
Log-rank test
p = 0.0006
Log-rank test
p = 0.0446
Progression-free survival (%)
Overall survival (%)
50
50
Late onset
Late onset
Early onset
Early onset
0
2.5
5
7.5
10
12.5
15
17.5
0
5
10
15
20
25
30
(months)
(months)
Late
Early
18 (0)
7 (0)
12 (0)
1 (0)
7 (0)
0 (0)
4 (0)
0 (0)
3 (0)
0 (0)
2 (0)
0 (0)
1 (0)
0 (0)
0 (1)
0 (0)
Late
Early
18 (0)
7 (0)
15 (0)
2 (0)
9 (0)
1 (0)
2 (1)
0 (0)
1 (1)
0 (0)
1 (0)
0 (0)
0 (1)
0 (0)
Number at risk (number censored)
Number at risk (number censored)
The influence of liver dysfunction on PFS (A) and OS (B) in patients with diverse cancer types after ICI treatment (adjusted data). (C, D) Comparison of PFS (C) and OS (D) based on early onset and late onset of liver dysfunction after ICI treatment (adjusted data).
(PFS, progression-free survival; OS, overall survival; ICI, immune checkpoint inhibitor)
